# Randomized Controlled Trials of Add-On Antidepressants in Schizophrenia

**DOI:** 10.1093/ijnp/pyv049

**Published:** 2015-06-12

**Authors:** Viacheslav Terevnikov, Grigori Joffe, Jan-Henry Stenberg

**Affiliations:** Kellokoski Hospital, Kellokoski, Finland (Dr Terevnikov); Department of Psychiatry, Helsinki University Central Hospital, Hospital District of Helsinki and Uusimaa, Helsinki, Finland (Drs Joffe and Stenberg).

**Keywords:** antidepressants, antipsychotics, schizophrenia, add-on treatment

## Abstract

**Background:**

Despite adequate treatment with antipsychotics, a substantial number of patients with schizophrenia demonstrate only suboptimal clinical outcome. To overcome this challenge, various psychopharmacological combination strategies have been used, including antidepressants added to antipsychotics.

**Methods:**

To analyze the efficacy of add-on antidepressants for the treatment of negative, positive, cognitive, depressive, and antipsychotic-induced extrapyramidal symptoms in schizophrenia, published randomized controlled trials assessing the efficacy of adjunctive antidepressants in schizophrenia were reviewed using the following parameters: baseline clinical characteristics and number of patients, their on-going antipsychotic treatment, dosage of the add-on antidepressants, duration of the trial, efficacy measures, and outcomes.

**Results:**

There were 36 randomized controlled trials reported in 41 journal publications (n=1582). The antidepressants used were the selective serotonin reuptake inhibitors, duloxetine, imipramine, mianserin, mirtazapine, nefazodone, reboxetin, trazodone, and bupropion. Mirtazapine and mianserin showed somewhat consistent efficacy for negative symptoms and both seemed to enhance neurocognition. Trazodone and nefazodone appeared to improve the antipsychotics-induced extrapyramidal symptoms. Imipramine and duloxetine tended to improve depressive symptoms. No clear evidence supporting selective serotonin reuptake inhibitors’ efficacy on any clinical domain of schizophrenia was found. Add-on antidepressants did not worsen psychosis.

**Conclusions:**

Despite a substantial number of randomized controlled trials, the overall efficacy of add-on antidepressants in schizophrenia remains uncertain mainly due to methodological issues. Some differences in efficacy on several schizophrenia domains seem, however, to exist and to vary by the antidepressant subgroups—plausibly due to differences in the mechanisms of action. Antidepressants may not worsen the course of psychosis. Better designed, larger, and longer randomized controlled trials are needed.

## Introduction

It is well established that antipsychotics are effective in the majority of patients with schizophrenia (Leucht et al., 2011). However, from one-fifth to one-third of the overall number of subjects undergoing the treatment demonstrate only partial, if any, improvement despite the antipsychotic treatment, adequate in terms of dosage and duration (Pantelis and Lambert, 2003). Treatment of these patients remains a major challenge, causing a serious burden for patients and their families and incurring high public health costs (Jablenski, 2000).

Clozapine, the prototypic “atypical” antipsychotic (presently referred to most often as second-generation antipsychotic [SGA]), is proven to be effective in a significant proportion of the patients who do not respond to other antipsychotic medications (Kane et al., 1998; Asenjo-Lobos et al., 2010; Kane and Correll, 2010). The mechanisms of the superior efficacy of clozapine are still obscure and are usually attributed to the drug’s complex receptor profile (Meltzer, 2012). However, some serious, sometimes life-threatening, adverse effects of clozapine (eg, weight gain, epileptic seizures, ileus, or agranulocytosis) limit its use in clinical practice (Kane et al., 1998). This calls for the search of new treatment strategies, including psychopharmacological approaches.

Indeed, a number of medications have been studied as adjuncts to antipsychotics with a goal to improve positive, negative, affective, or cognitive symptoms of schizophrenia resistant to antipsychotic medication alone. These pharmacological agents include lithium, anticonvulsants, antiinflammatory and glutamatergic drugs, sex hormones, cholinesterase and phosphodiesterase inhibitors, and various antidepressants (Singh et al., 2010; Leucht et al., 2011; Vernon et al., 2014).

Although the use of antidepressants added to antipsychotics in schizophrenia has been a subject of intensive research during the recent decades, the evidence regarding their efficacy still remains conflicting (Hinkelmann et al., 2013). Nevertheless, antidepressants tend to be routinely used by clinicians (Zink et al., 2010; Himelhoch et al., 2012). For instance, in the Clinical Trials of Intervention Effectiveness study, about one-third of the participants were receiving an antidepressant at the study baseline (Chakos et al., 2006). Thus, there seems to exist a gap between the wide use of antidepressants in clinical practice and the research evidence supporting this approach.

The present study aimed to review the published randomized controlled trials (RCTs) with antidepressants added to antipsychotics in the treatment of schizophrenia.

## Methods

Published RCTs assessing the efficacy of adjunctive antidepressants in schizophrenia were searched for in the PUBMED, PsycINFO, and PsycLIT databases from January 1960 to December 2013, using the following keywords: “schizophrenia” AND “antidepressant” OR “tricyclic antidepressant” OR “monoaminoxidase inhibitor” OR “selective serotonin reuptake inhibitor” OR “norepinephrine reuptake inhibitor”, as well as “schizophrenia” AND “amitriptyline” OR “imipramine” OR “clomipramine” OR “fluoxetine” OR “fluvoxamine” OR “sertraline” OR “paroxetine” OR “citalopram” OR “escitalopram” OR “venlafaxine” OR “duloxetine” OR “bupropion” OR “milnacipran” OR “reboxetine” OR “trazodone” OR “nefazodon” OR “mianserin” OR “mirtazapine” OR “vortioxetine” OR “vilazodone” OR “agomelatine”, as well as “schizophrenia” AND “double-blind” AND “augmentation”, as well as “schizophrenia” AND “double-blind” AND “adjunctive.” To obtain further data, hand searches of references in published review articles as well as cross-referencing were used.

All citations were reviewed using the following parameters: baseline clinical characteristics of patients and their antipsychotic treatment, dose of the add-on antidepressant, duration of the trial, number of participants, efficacy measures, and outcome.

## Results

We were able to locate a total of 36 RCTs (reported in 41 journal publications) including 1582 subjects with a diagnosis of schizophrenia or schizoaffective disorder (Table 1). All included trials employed head-to-head, parallel group, double-blind design comparing the efficacy of an add-on antidepressant vs add-on placebo with the exception of trials by Friedman et al. (2005) and Stryjer et al. (2010) that employed a crossover design. The vast majority of trials were small with the number of subjects ranging from 14 to 53. This number was exceeded only in the trial by Salokangas and coauthors (1996) (n=90). The duration of the trials ranged from 1 to 24 weeks. In 18 trials, an antidepressant was added to first-generation antipsychotics (FGAs), in 11 trials to SGAs, and in the rest of the trials to various antipsychotics.

**Table 1.** Antidepressants as Add-On Treatment in Schizophrenia: Randomized Placebo-Controlled Trials Table 1a. Antidepressants in Treatment of Negative and Positive Symptoms

**AntidepressantGroup**	**Author(s**)	**Year**	**Antidepressant**	**Dose,** **Mg**	**Antipsychotic**	**N**	**Duration,** **wk**	**Efficacy** **Measures**	**Outcome**
TCA	Collins and Dungas	1967	Amitryptiline	n/r	Perphenazine	87	12	Wing scale	Improvement in amitriptyline group.
	Waehrens and Gerlach	1980	Maprotiline	n/r	Various FGAs	20	16	n/r	No between group differences.
	Siris et al.	1991	Imipramine	n/r	Depot FGAs	14	24	n/r	Improvement in negative symptoms in imipramine group.
SSRI	Spina et al.	1994	Fluoxetine	20	Various FGAs	34	12	SANS, SAPS	Improvement on the SANS in fluoxetine group. No change on the SAPS in either group.
	Goff et al.	1995	Fluoxetine	20	Depot FGAs	41	6	BPRS	Greater improvement in fluoxetine group on the BPRS negative subscale. No change on the BPRS positive subscale in either group.
	Buchanan et al.	1996	Fluoxetine	20	Clozapine	33	8	SANS	No change in either group.
	Arango et al.	2000	Fluoxetine	20–40	Various FGAs	32	8	BPRS, SANS	No change in either group.
	Poyurovski et al.*	2002	Fluoxetine	20	Olanzapine	30	8	SAPS, SANS	Greater reduction on the SAPS in placebo group than in fluoxetine group.
	Bustillo et al.*	2003	Fluoxetine	60	Olanzapine	31	8	PANSS	No change in either group.
	Silver and Nassar	1992	Fluvoxamine	50–100	Various FGAs	30	7	SANS, SAPS	Improvement on the SANS in fluvoxamine group. No change on the SAPS in either group.
	Silver et al.	2000	Fluvoxamine	50–100	Various FGAs	53	6	SANS, SAPS	Improvement on the SANS in fluvoxamine group. No change on the SAPS in either group.
	Lee et al.	1998	Sertraline	50	Haloperidol	36	8	PANSS	No change in either group.
	Mulholand et al.*	2003	Sertraline	50	Variopus FGAs, risperidone	26	8	BPRS, SANS	No change in either group.
	Salokangas et al.	1996	Citalopram	20–40	Various FGAs	90	12	PANSS	Decrease on the PANSS total scale in both groups, no between group differences.
	Friedman et al.	2005	Citalopram	40	Various SGAs	19	24	PANSS	No change in either group.
	Iancu et al.	2010	Escitalopram	20	Various FGAs and SGAs	38	10	PANSS, SANS, CGI	No change in either group.
	Jockers et al.	2005	Paroxetine	30	Various FGAs and SGAs	29	12	PANSS	Improvement in paroxetine group on the PANSS negative subscale.
NRI	Schutz and Berk	2001	Reboxetine	8	Haloperidol	30	8	PANSS	No change in either group.
	Poyurovski et al.*	2003	Reboxetine	4	Olanzapine	26	6	SANS, SAPS	No change in either group.
SNRI	Mico et al.	2011	Duloxetine	60	Clozapine	33	16	BPRS, PANSS	Improvement on the BPRS, PANSS negative, PANSS general and PANSS total (sub)scales in duloxetine group.
NDRI	Evins et al.*	2005	Bupropion	300	Clozapine, FGAs, SGAs	53	12	SANS	No change in either group.
	Weiner et al.*	2012	Bupropion	300	Clozapine, FGAs, SGAs	32	12	n/a	No change in either group
Receptor- blocking antidepressants	Decina et al.	1994	Trazodone	n/r	n/r	47	6	BPRS, SANS	Improvement on the BPRS and SANS in trazodone group.
	Hayashi et al.	1997	Trazodone	50–200	n/r	39	5	BPRS, SANS	Improvement on the BPRS negative subscale in trazodone group.
	Stryjer et al.*	2010	Trazodone	100	n/r	13	1	PANSS	No change in either group.
	Shiloh et al.	2002	Mianserin	30	Various FGAs	18	6	BPRS, SAPS, SANS	Improvement on the BPRS in mianserin group.
	Hayashi et al.	1997	Mianserin	20–60	n/r	39	5	BPRS, SANS	Improvement on the BPRS and SANS in mianserin group.
	Poyurovski et al.	2003	Mianserin	15	Various FGAs	30	4	SAPS, SANS	No change in either group.
	Berk et al.	2001	Mirtazapine	30	Haloperidol	30	6	PANSS	Improvement on the PANSS negative subscale in mirtazapine group.
	Zoccali et al.	2004	Mirtazapine	30	Clozapine	24	8	SANS, SAPS, BPRS	Improvement on the SANS and BPRS general subscale in mirtazapine group.
	Joffe et al.	2009	Mirtazapine	30	Various FGAs	39	6	PANSS	Improvement on the PANSS total and all PANSS subscales in mirtazapine group.
	Berk et al.	2009	Mirtazapine	30	Various SGAs	40	6	PANSS	No change in either group.
	Abbasi et al.	2010	Mirtazapine	30	Risperidone	40	8	PANSS	Improvement on the PANSS negative and PANSS total in mirtazapine group.
	Cho et al.	2011	Mirtazapine	n/r	Risperidone	21	8	PANSS, SANS	Improvement on the PANSS negative and SANS in mirtazapine group.
	Caforio et al.	2013	Mirtazapine	30	Olanzapine	28	8	PANSS	Improvement on PANSS negative subscale in mirtazapine group.

**Table 1b.** Antidepressants in Treatment of Depressive Symptoms

**Antidepressant Group**	**Author(s**)	**Year**	**Antidepressant**	**Dose,** **mg**	**Antipsychotic**	**N**	**Duration,** **wk**	**Efficacy** **Measures**	**Outcome**
TCA	Siris et al.	2000	Imipramine	n/r	Flufenazine decanoate	70	6	HDRS, SADS	Improvement in imipramine group.
SSRIs	Spina et al.	1994	Fluoxetine	20	Various FGAs	34	12	HDRS	Improvement in fluoxetine group.
	Buchanan et al.	1996	Fluoxetine	20	Clozapine	33	8	n/r	No change in either group.
	Arango et al.	2000	Fluoxetine	20–40	Various FGAs	32	8	HDRS	No change in either group.
	Bustillo et al.*	2003	Fluoxetine	60	Olanzapine	31	8	HDRS	No change in either group.
	Mulholand et al.	2003	Sertraline	50	Various FGAs, risperidone	26	8	HDRS, BDI	Improvement on both measures in sertraline group.
	Jockers et al.	2005	Paroxetine	30	Various FGAs and SGAs	29	12	HDRS	No change in either group.
	Iancu et al.	2010	Escitalopram	20	Various FGAs and SGAs	38	10	HDRS	No change in either group.
NRI	Schutz and Berk	2001	Reboxetine	8	Haloperidol	30	8	HDRS	No change in either group.
	Poyurovski et al.*	2003	Reboxetine	4	Olanzapine	26	6	HDRS	Improvement in reboxetine group.
SNRI	Mico et al.	2011	Duloxetine	60	Clozapine	33	16	CDSS	Improvement in duloxetine group.
NDRI	Dufresne et al.	1988	Bupropion	300	FGAs	38	12	BPRS	Reduction of depressive symptoms in placebo group.
	Evins et al.*	2005	Bupropion	300	Clozapine, FGAs, SGAs	53	12	HDRS	No change in either group.
Receptor-blocking antidepressants	Shiloh et al.	2002	Mianserin	30	Haloperidol, perphenazine	18	6	HDRS	Improvement on the HDRS in both groups with no between-group difference.
	Poyurovski et al.	2003	Mianserin	15	Various FGAs	30	4	HDRS	No change in either group.
	Stryjer et al.*	2010	Trazodone	100	n/r	13	1	HDRS	No change in either group.
	Zoccali et al.	2004	Mirtazapine	30	Clozapine	24	8	BPRS (depressive factor)	No change in either group.
	Berk et al.	2009	Mirtazapine	30	Various SGAs	40	6	CDSS, HDRS	No change in either group.
	Terevnikov et al.	2011	Mirtazapine	30	Various FGAs	41	6	CDSS, PANSS (depression item)	Improvement on both measures in mirtazapine group.

**Table 1c.** Antidepressants in Treatment of Extrapyramidal Symptoms

**Antidepressant Group**	**Author(s**)	**Year**	**Antidepressant**	**Dose,** **mg**	**Antipsychotic**	**N**	**Duration,** **wk**	**Efficacy Measures**	**Outcome**
SSRIs	Goff et al.	1995	Fluoxetine	20	Depot FGAs	41	6	n/r	No change in either group.
	Arango et al.	2000	Fluoxetine	20–40	Various FGAs	32	8	MIMS	No change in either group.
	Bustillo et al.*	2003	Fluoxetine	60	Olanzapine	31	8	SAS, BAS, AIMS	No change in either group.
	Lee et al.	1998	Sertraline	50	Haloperidol	36	8	SAS	No change in either group.
	Mulholand et al.*	2003	Sertraline	50	Various FGAs, risperidone	26	8	ESRS, BAS	No change in sertraline group, worsening of ESRS scores in placebo group.
	Jockers et al.	2005	Paroxetine	30	Various FGAs and SGAs	29	12	SAS, BAS, AIMS	No change in either group.
	Iancu et al.	2010	Escitalopram	20	Various FGAs and SGAs	38	10	AIMS	No change in either group.
NRI	Schutz and Berk	2001	Reboxetine	8	Haloperidol	30	8	SAS	No change in either group.
	Poyurovski et al.*	2003	Reboxetine	4	Olanzapine	26	6	BAS, SAS	SAS scores decreased in both groups with no between-group difference in observed change.
Receptor-blocking antidepressants	Hayashi et al.	1997	Trazodone	50–200	n/r	39	5	AIMS	Improvement in trazodone group.
	Stryjer et al.	2010	Trazodone	100	n/r	13	1	SAS, BAS	Between group differences on the BAS scores in favour of trazodone. No changes on SAS scores in either group.
	Hayashi et al.	1997	Mianserin	20–60	n/r	39	5	AIMS	No change in either group.
	Poyurovski et al.	2003	Mianserin	15	Various FGAs	30	4	BAS, SAS, AIMS	No change in either group.
	Berk et al.	2001	Mirtazapine	30	Haloperidol	30	6	SAS	No change in either group.
	Joffe et al.*	2009	Mirtazapine	30	Various FGAs	39	6	SAS	Improvement on the SAS scores in mirtazapine group. No between group differences.
	Berk et al.	2009	Mirtazapine	30	Various SGAs	40	6	SAS	No change in either group.
	Abbasi et al.	2010	Mirtazapine	30	Risperidone	40	8	ESRS	No change in either group.
	Lee et al.	2011	Mirtazapine	15–30	Risperidone	21	8	SAS, BAS	No between group differences on either measure.
	Wynchank and Berk	2003	Nefazodone	100	Haloperidol	49	1	SAS, BAS, AIMS	Improvement on the SAS in nefazodone group. No changes on the BAS or AIMS in either group.

**Table 1d.** Antidepressants in Treatment of Cognitive Symptoms

**Author(s**)	**Year**	**Antidepressant**	**Dose,** **mg**	**Antipsychotic**	**N**	**Duration,** **wk**	**Neurocognitive parameters**	**Outcome**
Friedman et al.*	2005	Citalopram	40	Various SGAs	19	24	Attention, working memory, executive function	No change in either group.
Evins et al.*	2005	Bupropion	300	Clozapine, FGAs, SGAs	53	4	Attention, verbal learning and memory, working memory, executive function	Improvement in attention tests in the bupropion group.
Poyurovski et al.*	2003	Mianserin	15	Various FGAs	30	4	Learning, memory, sustained attention, executive function	Between group differences in favour of mianserin on memory and learning tests.
Stenberg et al.*	2010	Mirtazapine	30	Various FGAs	37	6	Visuo-spatial functions, verbal and visual memory, executive functions, verbal fluency, and general mental and psychomotor speed	Between group differences in favour of mirtazapine in visual-spatial ability and general mental speed/attentional control.

Abbreviations: AIMS, Abnormal Involuntary Movements Scale; BAS, Barnes Akathisia Scale; BDI, Beck Depression Inventory; BPRS, Brief Psychiatric Rating Scale; CDSS, Calgary Depression Scale for Schizophrenia; CGI, Clinical Global Impression scale; ESRS, Extrapyramidal Symptom Rating Scale; FGA, first-generation antipsychotic; HDRS, Hamilton Rating Scale for Depression; MADRS, Montgomery–Åsberg Depression Rating Scale; MIMS, Maryland Psychiatric Research Center Involuntary Movement Scale; NDRI, noradrenaline and dopamine reuptake inhibitor; n/r, not reported; NRI, selective noradrenaline reuptake inhibitor; PANSS, Positive and Negative Syndrome Scale; SADS, Scale for the Assessment of Depression and Schizophrenia; SANS, Scale for the Assessment of Negative Symptoms; SAPS, Scale for the Assessment of Positive Symptoms; SAS, Simpson-Angus Scale; SGA, second-generation antipsychotic; SNRI, selective serotonin and noradrenaline reuptake inhibitor; SSRI, selective serotonin reuptake inhibitor.

^*^ Studies in which the efficacy measure was a subject for secondary analysis.

In all trials, the efficacy measures—Brief Psychiatric Rating Scale, Positive and Negative Syndrome Scale (PANSS), Scale for the Assessment of Negative Symptoms, Scale for the Assessment of Positive Symptoms, Clinical Global Impression Scale, and the Wing’s scale—were used prospectively from baseline to endpoint. In only 14 studies were a priori defined minimum cut-off points on the Brief Psychiatric Rating Scale or PANSS scales used as an inclusion criterion, while in the others the subjects were vaguely defined to be “symptomatic” or “suffering from positive or negative symptoms” despite adequate antipsychotic treatment. Twenty-two trials included clinically stable subjects. Furthermore, in 21 trials, a thoroughly defined period of an unchanged antipsychotic treatment was required prior to participation in the study. This period varied from 1 week to 6 months. Presence of the current significant depressive symptoms served as an exclusion criterion in 12 trials. Of the others, 15 trials used depression rating scales as an outcome measure: the Hamilton Rating Scale for Depression, Calgary Depression Scale for Schizophrenia, Beck Depression Inventory, or Montgomery–Åsberg Depression Rating Scale. Extrapyramidal symptom rating scales—the Simpson-Angus Scale for extrapyramidal effects, the Abnormal Involuntary Movements Scale, and the Barnes Akathisia Scale—were used in 17 trials. Noteworthy, 5 trials were initially designed to investigate the effect of antidepressant add-on treatment of antipsychotic-induced weight gain (Poyurovski et al., 2002; Bustillo et al., 2003; Poyurovski et al., 2003a) or smoking cessation (Evins et al., 2005a; Weiner et al., 2012). These trials also utilized psychopathology rating scales (PANSS, Scale for the Assessment of Negative Symptoms, Scale for the Assessment of Positive Symptoms, Hamilton Rating Scale for Depression) and were thus included into this review.

The add-on antidepressant and add-on placebo groups did not generally differ significantly in baseline clinical characteristics. In only one trial (Joffe et al., 2009) the add-on antidepressant group had somewhat higher PANSS total scores at baseline.

### Add-On Antidepressants in the Treatment of Positive and Negative Symptoms of Schizophrenia

The included RCTs are presented in Table 1. In regard to the negative symptoms, trials with the tricyclic antidepressants (TCAs) yielded mainly positive results, but the studies were relatively old (Collins and Dundas, 1967; Waehrens and Gerlach, 1980; Siris et al., 1991). The outcomes of the SSRI trials as a group were controversial. Of 6 studies with fluoxetine, only 2 were positive (Spina et al., 1994; Goff et al., 1995). While both studies with fluvoxamine (Silver and Nassar, 1992; Silver et al., 2000) and the only study with paroxetine (Jockers-Scherubl et al., 2005) were positive, adjunctive citalopram (Salokangas et al., 1996; Friedman et al., 2005), escitalopram (Iancu et al., 2010), and sertraline (Lee et al., 1998; Mulholland et al., 2003) failed to demonstrate any benefits in any of the published trials. Likewise, both trials with the selective noradrenaline reuptake inhibitor reboxetine were negative (Schutz and Berk, 2001; Poyurovsky, 2003a). Conversely, in the only trial with the selective serotonin and noradrenaline reuptake inhibitor duloxetine, the active medication outperformed placebo on the PANSS negative subscale (Mico et al., 2011). Bupropion, a noradrenaline and dopamine reuptake inhibitor, demonstrated a trend toward improvement in negative symptoms that did not reach statistical significance in one RCT (Evins et al., 2005a). In another RCT (Weiner et al., 2012), bupropion did not override placebo (for review, see Englisch et al., 2013). For mianserine, trazodon, nefazodon, and mirtazapine (further referred to as receptor-blocking antidepressants, since they affect serotonin 5HT2 receptors rather than monoamine transporters, as opposed to the vast majority of existing antidepressants that are monoamine transporter blockers), most of the RCTs (8 of 12) were positive: 2 of 3 studies with trazodone (Decina et al., 1994; Hayashi et al., 1997), 1 of 3 studies with mianserin (Hayashi et al. 1997), and 6 of 7 studies with mirtazapine (Berk et al., 2001; Zoccali et al., 2004; Joffe et al., 2009; Abbasi et al., 2010; Cho et al., 2011; Caforio et al., 2013).

In only one study, an add-on antidepressant (mirtazapine) improved the positive symptoms of schizophrenia (Joffe et al., 2009). Noteworthy, a trend (*P*=.07) in favor of an antidepressant was also found in 1of 3 RCTs with mianserin (Shiloh et al., 2002).

No add-on RCTs with novel antidepressants agomelatine, vortioxetine, or vilazodone are yet available.

### Add-On Antidepressants in the Treatment of Depressive Symptoms of Schizophrenia

Antidepressant augmentation was found to be effective in the treatment of depressive symptoms of schizophrenia in only a small number of studies. These included imipramine (Siris et al., 1987), fluoxetine (Spina et al., 1994), sertraline (Mulholland et al., 2003), citalopram (Zisook, 2009), reboxetine (Poyurovski et al., 2003a), duloxetine (Mico et al., 2011), and mirtazapine (Terevnikov et al., 2011). The majority of the identified studies, however—3 RCTs with fluoxetine (Buchanan et al., 1996; Arango et al., 2000; Bustillo et al., 2003), 2 RCTs with mirtazapine (Zoccali et al., 2004; Berk et al., 2009), and one RCT for each with paroxetine (Jockers-Scherubl et al., 2005), reboxetine (Schutz and Berk, 2001), mianserin (Poyurovski et al., 2003b), and trazodone (Stryjer et al., 2010)—generated negative results. Also both RCTs with bupropion (Dufresne et al., 1988; Evins et al., 2005a) were negative (though a desired trend was observed in the latter one. For review, see Englisch et al. [2013]).

### Add-On Antidepressants in the Treatment of Extrapyramidal Side-Effects of Antipsychotics

All trials with the SSRIs (Goff et al., 1995; Lee et al., 1998; Arango et al., 2000; Bustillo et al., 2003; Mulholand et al., 2003; Jockers-Scherubl et al., 2005) demonstrated negative outcomes, as did both trials with reboxetine (Schutz and Berk, 2001; Poyurovksi et al., 2003a). Of the receptor-blocking antidepressants, mianserin failed to show superiority over placebo in both conducted trials (Hayashi et al., 1997; Poyurovski et al., 2003b), while both trials with trazodone (Hayashi et al., 1997; Stryjer et al., 2010) and the only trial with nefazodone (Wynchank and Berk, 2003) were positive. Mirtazapine was superior to placebo in alleviating antipsychotic-induced extrapyramidal symptoms (EPS) in only 1 of the 5 conducted trials (Joffe et al., 2009).

### Add-On Antidepressants in the Treatment of Cognitive Dysfunction in Schizophrenia

Of the SSRIs, the only available study (one with citalopram) did not reveal any improvement in cognitive functions (Friedman et al., 2005). Of the receptor-blocking antidepressants, mianserin added to FGAs showed beneficial effects on memory and learning with no between-group differences for executive functions (Poyurovski et al., 2003b). Mirtazapine outperformed placebo in between-group comparisons for visual-spatial ability and general mental speed/attentional control but not for other neurocognitive functions (Stenberg et al., 2010). Evins and collaborators (2005b) reported improvement in attention tests in an RCT with adjunctive bupropion, a noradrenaline and dopamine reuptake inhibitor.

## Results

The effects of different groups of add-on antidepressants on major psychopathology domains of schizophrenia are summarized in Table 2. The vast majority of publications reported no favorable effect of add-on antidepressants on the schizophrenia symptom domains. The only exception was the group of the receptor blocking antidepressants, with 9 studies reporting desirable results for negative symptoms (vs 3 studies that did not show benefits for this symptom domain) and, correspondingly, 2 studies (vs 1 study) for cognitive deficits.

**Table 2.** Effects of Different Groups of Add-On Antidepressants on Major Psychopathology Domains of Schizophrenia: Summary of Randomized Controlled Trials (RCTs)

**AntidepressantGroup**	**Effect**	**Negative Symptoms**	**Positive Symptoms**	**Depressive symptoms**	Extrapyramidal symptoms	Cognitive symptoms
TCA	+	1,4 (n=101)	n/a	4 (n=14)	n/a	n/a
	0	2 (n=20)	n/a	n/a	n/a	n/a
SSRI	+	5,7,8,15,29 (n=187)	n/a	7,21 (n=60)	n/a	n/a
	0	9,10,13,14,18,20,21,28,35 (n=335)	5,7,8,9,10,13,14,15,18,20,21,28,2 9 (n=489)	9,14,20,29,35 (n=163)	8,13,14,20,21,29,35 (n=233)	28 (n=19)
NRI	+	n/a	n/a	22	n/a	n/a
	0	17,22 (n=56)	17,22 (n=56)	17 (n=30)	17,22 (n=56)	n/a
SNRI	+	38 (n=33)	n/a	38 (n=33)	n/a	n/a
	0	n/a	38 (n=33)	n/a	n/a	n/a
NDRI	+	n/a	n/a	n/a	n/a	27 (n=53)
	0	26, 40 (n=85)	26, 40 (n=85)	3, 26 (n=91)	n/a	n/a
Receptor blocking antidepressants	+	6,11,12,16,19,25,31,32,36,41 (n=325)	31 (n=39)	39 (n=41)	11,24,31,34 (n=140)	23,33 (n=67)
	0	23,30,34 (n=83)	6,11,12,16,19,23,25,30,32,34,36 (n=341)	19,23,25,30,34 (n=125)	12,16,23,30,32,37 (n=200)	n/a

Abbreviations: n/a, no published RCTs found; NDRI, noradrenaline and dopamine reuptake inhibitor; NRI, selective noradrenaline reuptake inhibitor; SNRI, serotonin and noradrenaline reuptake inhibitor; SSRI, selective serotonin reuptake inhibitor; TCA, tricyclic antidepressant; +, result favoring antidepressant over placebo; 0, no difference between antidepressant and placebo.

Worsening of schizophrenia symptoms was not reported in any of the studies. The numbers in the syndrome columns refer to the index number of the RCT listed below. The list is arranged in chronological order, meaning that the larger the number the more recent (and often methodologically more reliable) is the corresponding study. The positive (+) and negative (0) studies ratio depicts roughly the balance of pro et contra evidence for each antidepressant group in each symptom domain. (The studies are not weighted in terms of methodological quality or number of participants and should be thus taken with caution).

No add-on RCTs with new novel antidepressants vortioxetine, vilazodone, or agomelatine could be located.

### Adverse Effects Due to Adjunctive Antidepressants in Schizophrenia

The majority of the included studies reported overall good tolerability of antidepressant-antipsychotic combinations. Spina et al. (1994) observed, however, more common adverse effects in patients receiving add-on fluoxetine compared with those receiving an antipsychotic alone. Noteworthy, no RCTs reported worsened psychosis due to adjunctive antidepressants.

## Discussion

We were able to identify 36 RCTs on combinations of antidepressants with an ongoing antipsychotic treatment published in the period from 1968 to 2013. The RCTs comprised the majority of the existing antidepressants and aimed to study their efficacy for the main clinical domains of schizophrenia. To summarize, the existing evidence regarding the efficacy of antidepressants in the treatment of schizophrenia is mixed.

The methodological quality of the studies varied. Overall, the later RCTs reported more precisely than the earlier ones the outcome criteria, the dosages of the study drugs, the clinical scales used, the patient population’s clinical and demographic characteristics, etc. This progress of the clinical study methodology advocates, in general, giving more weight to the most recent studies. Nevertheless, both old and recent RCTs have a number of restrictions. Almost all of them comprise small patient samples. Many publications express secondary analysis from the studies originally designed for negative (or sometimes also for overall) but not affective symptoms. This uncertainty possibly contributes to the heterogeneity of results obtained in different studies and makes a between-study comparison difficult.

### Antidepressants in the Treatment of Negative Symptoms of Schizophrenia

Only 5 of 14 RCTs with SSRIs reported favorable results. This puts into question the efficacy of this antidepressant group in the treatment of schizophrenia’s negative symptoms. From a theoretical viewpoint, these findings possibly indicate that the increase of serotonin availability in the brain per se is not enough to alleviate the negative symptoms.

The data on selective serotonin and noradrenaline reuptake inhibitor (SNRIs) are scarce. Only one small study (with duloxetine) is available. Its desirable result needs further validation in larger studies. Studies with other SNRIs are also necessary.

For the TCAs, even though 2 of the 3 existing studies were positive, the small number of trials and methodological issues (omitted report on outcome measures or use of psychiatric scales with questionable reliability) makes the significance of these results uncertain.

In both available studies with reboxetine and bupropion, the active drug was not more efficacious than placebo, indicating that catecholamine reuptake inhibition alone is seemingly insufficient to alleviate negative symptoms of schizophrenia.

Data on the receptor-blocking antidepressants are more convincing, since they outperformed placebo in 10 of the 13 reported RCTs. This advantage may plausibly be interpreted with the dissimilar pharmacodynamic mechanisms of action of these compounds. While not affecting monoamine transporters (and thus reuptake of monoamines), all receptor-blocking antidepressants inhibit the postsynaptic serotonin 5HT2 receptors, a property that they share with clozapine and other SGAs (Meltzer, 1999). The theory of atypicality, especially popular in the late 1990s, postulates that antipsychotics with higher affinity to the 5HT2 than to the dopamine D2 receptors (the atypical antipsychotics, or SGAs) were more effective in treating positive and negative symptoms and cognitive deficits and less prone to cause extrapyramidal side-effects compared with the FGAs, potent dopamine D2 blockers with low or negligible 5HT2 receptor inhibition (Meltzer and Massey, 2011). The rationale for the use of the receptor-blocking antidepressants in schizophrenia is the presumption that combination of an inhibitor of the 5HT2 receptor (antidepressant) with a relatively pure D2 blocker (FGA) would result in a clinical effect resembling that of the SGAs, with possible additional benefits in terms of efficacy and tolerability (Berk et al., 2001; Joffe et al., 2009). Preliminary evidence for this presumption was gained in a study by Duinkerke and coauthors (1993) in which add-on ritanserin, a pure 5HT2 blocker devoid of antidepressive properties, improved negative symptoms in haloperidol-treated schizophrenia patients.

On the other hand, in 4 of 6 positive studies, mirtazapine was added to risperidone, clozapine, or olanzapine –each of them known to be a potent 5HT2A/C blocker by itself, which makes little sense from the viewpoint of the above-mentioned atypicality theory. A possible explanation for this advantageous effect may be the affinity of mirtazapine to some other (than 5HT2) types of receptors. In addition to the 5HT2 receptors, mirtazapine inhibits the postsynaptic serotonin 5HT3 receptors and presynaptic noradrenaline α2 receptors and indirectly stimulates postsynaptic serotonin 5HT1A receptors (de Boer, 1996), all of these believed to be involved in the pathophysiology of schizophrenia. Antagonists of the 5HT3 receptors seem potentially beneficial in the treatment of schizophrenia (Costall and Naylor, 1992). In a preclinical study by Pitsikas and Borsini (1996), a 5HT3 receptor antagonist, itasetron, demonstrated procognitive effects, and in a clinical trial by Zhang and collaborators (2006), another 5HT3 antagonist, ondansetron, was added to an on-going treatment with haloperidol and improved negative symptoms, general psychopathology, and cognitive functions in patients with schizophrenia.

Blockade of the noradrenaline α2 receptors also may be useful in the treatment of schizophrenia. In an RCT by Litman and coauthors (1996), the α2 antagonist idazoxan added to fluphenazine produced clinical improvement comparable with that of clozapine. This finding was later supported by 2 preclinical studies. Wadenberg and coauthors (2007) found idazoxan to potentiate the antipsychotic-like effect of both FGA (haloperidol) and SGA (olanzapine) and also to reverse haloperidol-induced catalepsy in the laboratory animals. In another preclinical study, Marcus and coauthors (2010) reported that adjunctive idazoxan had improved the efficacy of risperidone and facilitated cortical dopaminergic and glutamatergic neurotransmission.

Rummel et al. (2005) performed a systematic review of 5 RCTs (comprising the TCAs, receptor-blocking antidepressants, and SSRIs) available at that time and fulfilling the rigorous systematic review criteria. The authors concluded that “the combination of antipsychotics and antidepressants may be more effective in treating negative symptoms of schizophrenia than antipsychotics alone.” This conclusion, although not differentiating between effects of different antidepressant subgroups, is mainly in line with that of the current review.

The efficacy of add-on antidepressants on negative symptoms of schizophrenia was reviewed by Singh and coauthors (2010). Based on an analysis of 23 RCTs (n = 819), the authors found that add-on treatment with antidepressants was more effective for negative symptoms in chronic schizophrenia than monotherapy with antipsychotics. In particular, Singh and collaborators concluded that fluoxetine, trazodone, and ritanserin were the most efficient antidepressants for treating negative symptoms, while the majority of other antidepressants did not express such advantages compared with placebo. These findings seem to be controversial, especially for fluoxetine, which has been unable to outperform placebo in 3 of 5 studies published to date. Unlike Singh and coworkers (2010), we have found that data supporting clinical efficacy of add-on antidepressants on negative symptoms is most consistent for the receptor-blocking antidepressants. This difference in the results may be partly explained by the different number of studies included in these 2 reviews (9 RCTs on receptor-blocking antidepressants in the review by Singh and co-authors vs 13 RCTs in the current review). Some of the RCTs with receptor-blocking antidepressants (Abbasi et al., 2010; Stryjer et al., 2010; Cho et al., 2011; Caforio et al., 2013) were not available at the time of preparation of the review by Singh and co-authors. Furthermore, the discrepancy could result from different methodology employed by Singh and co-authors, who based their conclusions on the computation of the effect size for each antidepressant separately.

Englisch and colleagues (2013) did not find in their review any RCT data on improved negative symptoms with adjunctive bupropion.

### Antidepressants in the Treatment of Positive Symptoms of Schizophrenia

Antidepressants seem to have no desirable effect on positive symptoms of schizophrenia, as almost all RCTs were disappointing in this respect. The only exception was a study by Joffe and coauthors (2009), in which mirtazapine (but not placebo) added to on-going therapy with various FGAs improved positive symptoms. This result diverged from other mirtazapine trials. It is possible that in that particular study, the researchers succeeded in minimizing the regression to the mean by a sound process of stabilization of clinical status and dosage of the FGAs. Also, placebo response and low adherence to the protocol procedures were minimized by a placebo run-in period, which probably explained the high patient retention level. Stabilization and low drop-out rate might permit for surfacing a possible additive antipsychotic effect of mirtazapine. This observation, however, has to be replicated in other studies with similarly rigorous methodology.

In clinical practice, physicians are sometimes concerned with a risk of worsening or exacerbation of psychosis when prescribing antidepressants to patients with schizophrenia (Petit, 1994). Moreover, some current schizophrenia treatment guidelines suggest caution when using antidepressants (APA, 2004). This review does not support this opinion, since not a single study with an add-on antidepressant reported worsening of positive symptoms. The majority of the presented RCTs, however, involved subjects with chronic schizophrenia, with only 3 trials comprising acutely ill subjects. It therefore remains unclear to what extent this observation can be extrapolated to acute schizophrenia.

### Antidepressants in the Treatment of Depressive Symptoms of Schizophrenia

Only 2 of 6 studies with SSRIs were positive, and exactly the same proportion (2 of 6) was found with regard to receptor-blocking antidepressants. This puts the efficacy of both antidepressant groups into question. The antidepressants with sound noradrenaline activity may, however, be of interest for this symptom domain, since 1 of the 2 existing RCTs with reboxetine, the only study with imipramine, and the only study with duloxetine (compounds with a pronounced noradrenaline enhancing effect) reported improved depression. A trend in favor of active compound over placebo was observed in 1 of 2 RCTs with adjunctive noradrenaline and dopamine inhibitor bupropion. Of the 6 published trials with the receptor-blocking antidepressants, only one (Terevnikov et al., 2011) reported improved depression with add-on mirtazapine.

To summarize, the evidence regarding the efficacy of antidepressants in the treatment of depression in schizophrenia is scarce and controversial. The main methodological issue in this area is that the study population was not selected specifically for depressive symptoms, with the only exception of the postpsychotic depression study by Siris and co-authors (1987). In the rest of the studies, change in depression scores was a subject for a secondary analysis.

Also, Whitehead and coauthors (2012) in their Cochrane review concluded that, although antidepressants may be of some benefit for people with depression and schizophrenia, the evidence was still far from convincing. The authors of that review also emphasized poor quality of the available literature as a contributing factor for this uncertainty.

### Antidepressants in the Treatment of Extrapyramidal Side-Effects of Antipsychotics

For the monoamine transporter inhibitors, only SSRIs and the noradrenaline reuptake inhibitor reboxetine studies were identified. All these studies were negative, probably not surprisingly given the lack of theoretical basis for such combination in EPS. Moreover, the SSRIs are reported to even cause EPS in patients with major depressive disorder (Govoni et al., 2001).

For the receptor-blocking antidepressants, a positive effect on the FGA-induced EPS was found in both studies with trazodone (Hayashi et al., 1997; Stryjer et al., 2010) but in neither of the mianserin studies (Hayashi et al., 1997; Poyurovski et al., 2003b). Also, 4 of 5 studies with mirtazapine (Berk et al., 2001, 2009; Abbasi et al., 2010; Lee et al., 2011) were negative. Nefazodone was found to improve haloperidol-induced Parkinsonism but not akathisia or tardive dyskinesia (Wynchank and Berk, 2003).

The use of receptor-blocking antidepressants for the EPS arises from the theoretical assumption of dopamine insufficiency in the basal ganglia as the cause of EPS (Tatara et al., 2012). Inhibition of the 5HT2A/C receptors—a common pharmacological effect of SGAs and receptor-blocking antidepressants—may increase availability of dopamine in this area, thereby alleviating EPS. Another possible mechanism of alleviating antipsychotic-induced EPS by some of the receptor-blocking antidepressants may be an indirect stimulation of the serotonin 5HT1A receptors that leads to decrease of serotonin at 5HT2C receptors located on dopaminergic neurons (Haleem, 2006).

In sum, despite encouraging theoretical presumptions, the evidence regarding the efficacy of receptor-blocking antidepressants for antipsychotic-induced EPS is limited and contradictory. Only trazodone and nefazodone were found effective, while mianserin and mirtazapine were probably not. As marketing of nefazodone has been discontinued, the nefazodone-related findings are no longer relevant for clinical practice. Moreover, positive findings with receptor-blocking antidepressants are available for only FGA-induced EPS and may be extrapolated on the SGAs only with caution. Given that the use of FGAs has been markedly diminished during recent years and further decrease may happen in the future, the results of these studies are rather of theoretical than of practical interest.

### Antidepressants in the Treatment of Cognitive Deficits Related to Schizophrenia

Neurocognitive effects of antidepressants added to antipsychotics in schizophrenia have been studied in only 3 RCTs.

A serotonin reuptake inhibitor, citalopram, did not improve cognition in schizophrenia (Friedman et al., 2005). The increase of availability of serotonin alone in the brain may not therefore improve neurocognition.

Evins and coauthors (2005b) reported improved attention in patients with schizophrenia treated with bupropion for smoking cessation. This positive change became significant only after controlling for abstinence status. The influence of nicotine use and smoking cessation on cognitive performance in this study makes the results difficult to interpret.

On the contrary, 2 receptor-blocking antidepressants with a similar mechanism of action, mianserin (Poyurovsky et al., 2003b) and mirtazapine (Stenberg et al., 2010, 2011), did improve neurocognition in FGA-treated schizophrenia patients.

As for the negative symptoms, the plausible proneurocognitive effect of mirtazapine and mianserin in schizophrenia may emerge from their receptor-binding profile. First, they may enhance prefrontal catecholamine activity via 5HT2A or 5HT2C receptor blockade (as most SGAs do), thereby enhancing activity of the prefrontal cortex (Liegeois et al., 2002; Menzies, 2007). Second, the 5HT3 receptor modulation by mianserin or mirtazapine could improve neurocognition (Akhondzadeh et al., 2009) through increased release of acetylcholine (Passani and Blandina, 1998). Third, mirtazapine might improve neurocognition as a result of the indirect agonism of 5HT1A receptors (Sumiyoshi et al., 2007). Fourth, mianserin and mirtazapine inhibit the noradrenergic α2 receptors. Inhibition of the α2 receptors can enhance neurocognition via noradrenaline-mediated modulation of response to environmental stimuli (Friedman et al., 2004). Furthermore, the α2 receptor antagonism seems to boost hippocampal neurogenesis (Rizk et al., 2006). Mirtazapine is an even more potent α2 antagonist than clozapine, which may explain its additional neurocognition-enhancing effect even if added to clozapine, as demonstrated in an open label trial by Delle Chiaie et al. (2007). Mirtazapine may also boost the levels of the Brain-Derived Neurotrophic Factor (Rogoz et al. 2005), a major mediator of neurogenesis and neuroplasticity, which are often abnormal in patients with schizophrenia (Rizos et al., 2008).

Vernon and coauthors (2014) found in their meta-analysis clinically negligible (although statistically significant) improvement of neurocognitive functions with adjuvant antidepressants. However, while Vernon and colleagues analyzed pooled antidepressants, in the current review, each antidepressant study has been given separately.

The number of studies presented in both reviews was small. Thus, the evidence for the proneurocognitive potential of add-on antidepressants is, so far, insufficient but deserves further research.

### Limitations

The RCT is the gold standard of clinical efficacy studies. However, RCTs are fraught with patient selection bias and, thus, the results of available naturalistic prospective studies, mirror design studies, as well as pertinent register studies should be taken into account for balanced clinical decisions. Furthermore, we have not performed quantitative analyses of the data nor included unpublished data as it would be required for a systematic meta-analysis. Moreover, the vast majority of the available RCTs are small and methodologically heterogenic, which calls for caution when interpreting our results. In a substantial part of the RCTs in this review, the reported outcomes were a result of secondary analysis.

This review has focused predominantly on the efficacy of adjunctive antidepressants, while in clinical practice the potential benefits should be balanced against unwanted side-effects of these drugs and of antidepressant-antipsychotic combinations.

### Further Directions

In the future, well-designed, larger, and longer RCTs are needed. There is also a need for trials designed specifically for certain symptom domains, with appropriately selected patients and pertinent rating scales. Despite an existing theoretical rationale, there is a lack of studies assessing the effects of bupropion, reboxetine, SNRIs, and reversible inhibitors of monoamine oxidase A on cognitive function in schizophrenia.

The future studies should also focus on new, novel, add-on antidepressants that have come into clinical practice in recent years. Of interest may be, for instance, vortioxetine. In addition to the conventional inhibition of the serotonin (5HT) transporter, vortioxetine demonstrates an affinity to the serotonin 5HT1a, 5HT1b, 5HT1d, 5HT3, and 5HT7 receptors. Furthermore, it modulates the GABA, MNDA, noradrenergic, dopaminergic, cholinergic, and histaminergic neurotransmitter systems (Leiser et al., 2015; Stahl, 2015). Some of these receptors and neurotransmitters may be involved in pathophysiology of schizophrenia. In addition to the symptoms of depression, vortioxetine may improve cognitive functions in patients with major depressive disorder (Sole et al., 2015).

## List of RCTs:

1. Collins and Dungas, 1967
2. Waehrens and Gerlach,1980
3. Dufresne et al., 1988
4. Siris et al., 1991
5. Silver and Nassar, 1992
6. Decina et al., 1994
7. Spina et al., 1994
8. Goff et al., 1995
9. Buchanan et al., 1996
10. Salokangas et al., 1996
11. Hayashi et al., 1997(a)
12. Hayashi et al., 1997(b)
13. Lee et al., 1998
14. Arango et al., 2000
15. Silver et al., 2000
16. Berk et al., 2001
17. Schutz and Berk, 2001
18. Poyurovski et al., 2002
19. Shiloh et al., 2002
20. Bustillo et al., 2003
21. Mulholand et al., 2003
22. Poyurovski et al., 2003(a)
23. Poyurovski et al., 2003(b)
24. Wynchank and Berk, 2003
25. Zoccali et al., 2004
26. Evins et al., 2005(a)
27. Evins et al., 2005(b)
28. Friedman et al., 2005
29. Jockers-Scherubl et al., 2005
30. Berk et al., 2009
31. Joffe et al., 2009
32. Abbasi et al., 2010
33. Stenberg et al., 2010
34. Stryjer et al. 2010
35. Iancu et al. 2010
36. Cho et al., 2011
37. Lee et al., 2011
38. Mico et al., 2011
39. Terevnikov et al., 2011
40. Weiner et al., 2012
41. Caforio et al., 2013

## Conclusions

There exists a substantial number of RCTs on the efficacy of add-on antidepressants, but due to methodological issues the results remain uncertain. Receptor-blocking antidepressants mirtazapine and mianserin show somewhat consistent efficaciousness for negative symptoms. The evidence for EPS, neurocognition, and depression domains is even more ambiguous and should be interpreted with caution. Some receptor-blocking antidepressants (trazodone and nefazodone) seem to improve FGA-induced EPS, while others (mirtazapine and mianserin) appear to enhance neurocognition. Transporter-blocking antidepressants with prominent noradrenergic activity tended to improve depressive symptoms.

The overall data for the SSRIs are rather disappointing. An add-on antidepressant (mirtazapine) improved positive symptoms in only one study. However, the data unequivocally show that adjunctive antidepressants do not worsen psychosis, at least in chronic schizophrenia. The caution recommended for antidepressants in some current schizophrenia treatment guidelines may, therefore, need reappraisal.

## Interest Statement

None.

## References

[CIT0001] Abbasi SH Behpournia H Ghoreshi A Salehi B Raznahan M Rezazadeh SA Rezaei F Akhondzadeh S (2010) The effect of mirtazapine add-on therapy to risperidone in the treatment of schizophrenia: a double-blind randomized placebo-controlled trial. Schizophr Res 116:101–106. 19959338 10.1016/j.schres.2009.11.008

[CIT0002] Akhondzadeh S Mohammadi N Noroozian M Karamghadiri N Ghoreishi A Jamshidi AH Forghani S (2009) Added ondansetron for stable schizophrenia: a double blind, placebo controlled trial. Schizophr Res 107:206–212. 18789844 10.1016/j.schres.2008.08.004

[CIT0003] American Psychiatric Association: Practice guidelines for the treatment of patients with schizophrenia, second edition (2004). Am J Psychiatry 161:1–56. 15000267

[CIT0004] Arango C Kirkpatrick B Buchanan RW (2000) Fluoxetine as an adjunct to conventional antipsychotic treatment of schizophrenia patients with residual symptoms. J Nerv Ment Dis 188:50–53. 10665462 10.1097/00005053-200001000-00010

[CIT0005] Asenjo Lobos C Komossa K Rummel-Kluge C Hunger H Schmid F Schwarz S Leucht S (2010) Clozapine versus other atypical antipsychotics for schizophrenia. Cochrane Database Syst Rev 10:CD006633. 10.1002/14651858. 10.1002/14651858.CD006633.pub2 PMC4169186 21069690

[CIT0006] Berk M Ichim C Brook S (2001) Efficacy of mirtazapine add on therapy to haloperidol in the treatment of the negative symptoms of schizophrenia: a double-blind randomized placebo-controlled study. Int Clin Psychopharmacol 16:87–92. 11236073 10.1097/00004850-200103000-00003

[CIT0007] Berk M Gama CS Sundram S Hustig H Koopowitz L D’Souza R Malloy H Rowland C Monkhouse A Monkhouse A Bole F Sathiyamoorthy S Piskulic D Dodd S (2009) Mirtazapine add-on therapy in the treatment of schizophrenia with atypical antipsychotics: a double-blind, randomised, placebo-controlled clinical trial. Hum Psychopharmacol 24:233–238. 19330802 10.1002/hup.1017

[CIT0008] Buchanan RW Kirkpatrick B Bryant N Ball P Breier A (1996) Fluoxetine augmentation of clozapine treatment in patients with schizophrenia. Am J Psychiatry 153:1625–1627. 8942462 10.1176/ajp.153.12.1625

[CIT0009] Bustillo JR Lauriello J Parker K Hammond R Rowland L Bogenschutz M Keith S (2003) Treatment of weight gain with fluoxetine in olanzapine-treated schizophrenic outpatients. Neuropsychopharmacol 28:527–529. 10.1038/sj.npp.1300089 12629532

[CIT0010] Caforio G Di Giorgio A Rampino A Rizzo M Romano R Taurisano P Fazio L De Simeis G Ursini G Blasi G Nardini M Mancini M Bertolino A (2013) Mirtazapine add-on improves olanzapine effect on negative symptoms of schizophrenia. J Clin Psychopharmacol 33:810–812. 24113675 10.1097/JCP.0b013e3182a4ec77

[CIT0011] Chakos MH1 Glick ID Miller AL Hamner MB Miller DD Patel JK Tapp A Keefe RS Rosenheck RA (2006) Baseline use of concomitant psychotropic medications to treat schizophrenia in the CATIE trial. Psychiatr Serv 57:1094–1101. 16870959 10.1176/ps.2006.57.8.1094

[CIT0012] Cho SJ Yook K Kim B Choi TK Lee KS Kim YW Lee JE Suh S Yook KH Lee SH (2011) Mirtazapine augmentation enhances cognitive and reduces negative symptoms in schizophrenia patients treated with risperidone: a randomized controlled trial. Prog Neuropsychopharmacol Biol Psychiatry 35:208–211. 21095214 10.1016/j.pnpbp.2010.11.006

[CIT0013] Collins AD Dundas J (1967) A double-blind trial of amitriptyline/perphenazine, perphenazine and placebo in chronic withdrawn inert schizophrenics. Br J Psychiatry 113:1425–1429. 4865295 10.1192/bjp.113.505.1425

[CIT0014] Costall B Naylor RJ (1992) Astra Award Lecture. The psychopharmacology of 5-HT3 receptors. Pharmacol Toxicol 71:401–415. 1362267 10.1111/j.1600-0773.1992.tb00570.x

[CIT0015] De Boer T (1996) The pharmacologic profile of mirtazapine. J Clin Psychiatry 57 (Suppl 4):19–25. 8636062

[CIT0016] Decina P Mukherjee S Bocola V Saraceni F Hadjichristos C Scapicchio P (1994) Adjunctive trazodone in the treatment of negative symptoms of schizophrenia. Hosp Community Psychiatry 45:1220–1223. 7868106 10.1176/ps.45.12.1220

[CIT0017] Delle Chiaie R Salviati M Fiorentini S Biondi M (2007) Add-on mirtazapine enhances effects on cognition in schizophrenic patients under stabilized treatment with clozapine. Exp Clin Psychopharmacol 15:563–568. 18179309 10.1037/1064-1297.15.6.563

[CIT0018] Dufresne RL Kass DJ Becker RE (1988) Bupropion and thiothixene versus placebo and thiothixene in the treatment of depression in schizophrenia. Drug Develop Res 12:259–266.

[CIT0019] Duinkerke SJ Botter PA Jansen AA van Dongen PA van Haaften AJ Boom AJ van Laarhoven JH Busard HL (1993) Ritanserin, a selective 5-HT2/1C antagonist, and negative symptoms in schizophrenia. A placebo-controlled double-blind trial. Br J Psychiatry 163:451–455. 7902766 10.1192/bjp.163.4.451

[CIT0020] Englisch S Morgen K Meyer-Lindenberg A Zink M (2013). Risks and benefits of bupropion treatment in schizophrenia: a systematic review of the current literature. Clin Neuropharmacol 36:203–215. 24201231 10.1097/WNF.0b013e3182a8ea04

[CIT0021] Evins AE Cather C Deckersbach T Freudenreich O Culhane MA Olm-Shipman CM Henderson DC Schoenfeld DA Goff DC Rigotti NA (2005a) A double-blind placebo-controlled trial of bupropion sustained-release for smoking cessation in schizophrenia. J Clin Psychopharmacol 25:218–225. 15876899 10.1097/01.jcp.0000162802.54076.18

[CIT0022] Evins AE Deckersbach T Cather C (2005b) Independent effects of tobacco abstinence and bupropion on cognitive function in schizophrenia. J Clin Psychiatry 66:1184–1190. 16187778 10.4088/jcp.v66n0915

[CIT0023] Friedman JI Stewart DG Gorman JM (2004) Potential noradrenergic targets for cognitive enhancement in schizophrenia. CNS Spectr 9:350–355. 15115947 10.1017/s1092852900009330

[CIT0024] Friedman JI Ocampo R Elbaz Z Parrella M White L Bowler S Davis KL Harvey PD (2005) The effect of citalopram adjunctive treatment added to atypical antipsychotic medications for cognitive performance in patients with schizophrenia. J Clin Psychopharmacol 25:237–242. 15876902 10.1097/01.jcp.0000161499.58266.51

[CIT0025] Goff DC Midha KK Sarid-Segal O (1995) A placebo-controlled trial of fluoxetine added to neuroleptic in patients with schizophrenia. Psychopharmacology (Berl) 117:417–423. 7604142 10.1007/BF02246213

[CIT0026] Govoni S Racchi M Masoero E Zamboni M Ferini-Strambi L (2001) Extrapyramidal symptoms and antidepressant drugs: neuropharmacological aspects of a frequent interaction in the elderly. Mol Psychiatry 6:134–142. 11317214 10.1038/sj.mp.4000801

[CIT0027] Haleem DJ (2006) Serotonergic modulation of dopamine neurotransmission: a mechanism for enhancing therapeutics in schizophrenia. J Coll Physicians Surg Pak 16:556–562. 16899192

[CIT0028] Hayashi T Yokota N Takahashi T Tawara Y Nishikawa T Yano T Furutani M Fujikawa T Horiguchi J Yamawaki S (1997) Benefits of trazodone and mianserin for patients with late-life chronic schizophrenia and tardive dyskinesia: an add-on, double-blind, placebo-controlled study. Int Clin Psychopharmacol 12:199–205. 9347380 10.1097/00004850-199707000-00003

[CIT0029] Himelhoch S Slade E Kreyenbuhl J Medoff D Brown C Dixon L (2012) Antidepressant prescribing patterns among VA patients with schizophrenia. Schizophr Res 136:32–35. 22325077 10.1016/j.schres.2012.01.008

[CIT0030] Hinkelmann K Yassouridis A Kellner M Jahn H Wiedemann K Raedler TJ (2013) No effects of antidepressants on negative symptoms in schizophrenia. J Clin Psychopharmacol 33:686–690. 23857309 10.1097/JCP.0b013e3182971e68

[CIT0031] Iancu I Tschernihovsky E Bodner E Piconne AS Lowengrub K (2010) Escitalopram in the treatment of negative symptoms in patients with chronic schizophrenia: a randomized double-blind placebo-controlled trial. Psychiatry Res 179:19–23. 20472299 10.1016/j.psychres.2010.04.035

[CIT0032] Jablensky A (2000) Epidemiology of schizophrenia: the global burden of disease and disability. Eur Arch Psychiatry Clin Neurosci 250:274–285. 11153962 10.1007/s004060070002

[CIT0033] Jockers-Scherubl MC Bauer A Godemann F Reischies FM Selig F Schlattmann P (2005) Negative symptoms of schizophrenia are improved by the addition of paroxetine to neuroleptics: a double-blind placebo-controlled study. Int Clin Psychopharmacol 20:27–31. 15602113 10.1097/00004850-200501000-00006

[CIT0034] Joffe G Terevnikov V Joffe M Stenberg JH Burkin M Tiihonen J (2009) Add-on mirtazapine enhances antipsychotic effect of first generation antipsychotics in schizophrenia: a double-blind, randomized, placebo-controlled trial. Schizophr Res 108:245–251. 19144501 10.1016/j.schres.2008.12.002

[CIT0035] Kane JM Honigfeld G Singer J Meltzer HY, Clozaril Collaborative Study Group (1998) Clozapine for the treatment-resistant schizophrenic. Arch Gen Psychiatry 45:789–796. 3046553 10.1001/archpsyc.1988.01800330013001

[CIT0036] Kane JM Correll CU (2010) Pharmacologic treatment of schizophrenia. Dialogues Clin Neurosci 12:345–357. 20954430 10.31887/DCNS.2010.12.3/jkane PMC3085113

[CIT0037] Lee MS Kim YK Lee SK Suh KY (1998) A double-blind study of adjunctive sertraline in haloperidol-stabilized patients with chronic schizophrenia. J Clin Psychopharmacol 18:399–403. 9790158 10.1097/00004714-199810000-00008

[CIT0038] Lee J Cho SJ Lee KS Yook K Choe AY Lee S Kim B Kim KH Choi TK Lee SH (2011) The tolerability of mirtazapine augmentation in schizophrenic patients treated with risperidone: a preliminary randomized placebo-controlled trial. Clin Psychopharmacol Neurosci 9:73–77. 23431108 10.9758/cpn.2011.9.2.73 PMC3569079

[CIT0039] Leiser SC Li Y Pehrson AL Dale E Smagin G Sanchez C (2015). Serotonergic regulation of prefrontal cortical circuitries involved in cognitive processing: a review of individual 5-HT receptor mechanisms and concerted effects of 5-HT receptors exemplified by the multimodal antidepressant vortioxetine. ACS Chem Neurosci. 2015 Mar 16. [Epub ahead of print] 10.1021/cn500340j 25746856

[CIT0040] Leucht S Heres S Kissling W Davis JM (2011) Evidence-based pharmacotherapy of schizophrenia. Int J Neuropsychop 14:269–284. 10.1017/S1461145710001380 21208500

[CIT0041] Liégeois JF Ichikawa J Meltzer HY (2002) 5-HT(2A) receptor antagonism potentiates haloperidol-induced dopamine release in rat medial prefrontal cortex and inhibits that in the nucleus accumbens in a dose-dependent manner. Brain Res 947:157–165. 12176156 10.1016/s0006-8993(02)02620-3

[CIT0042] Litman RE Su TP Potter WZ Hong WW Pickar D (1996) Idazoxan and response to typical neuroleptics in treatment-resistant schizophrenia. Comparison with the atypical neuroleptic, clozapine. Br J Psychiatry 168:571–579. 8733795 10.1192/bjp.168.5.571

[CIT0043] Marcus MM Wiker C Frånberg O Konradsson-Geuken A Langlois X Jardemark K Svensson TH (2010) Adjunctive alpha2-adrenoceptor blockade enhances the antipsychotic-like effect of risperidone and facilitates cortical dopaminergic and glutamatergic, NMDA receptor-mediated transmission. Int J Neuropsychopharmacol 13:891–903. 19835668 10.1017/S1461145709990794

[CIT0044] Meltzer HY (1999) Treatment of schizophrenia and spectrum disorders: pharmacotherapy, psychosocial treatments, and neurotransmitter interactions. Biol Psychiatry 46:1321–1327. 10578448

[CIT0045] Meltzer HY Massey BW (2011) The role of serotonin receptors in the action of atypical antipsychotic drugs. Curr Opin Pharmacol 11:59–67. 21420906 10.1016/j.coph.2011.02.007

[CIT0046] Meltzer HY (2012) Update on typical and atypical antipsychotic drugs. Annu Rev Med 64:1–6. 23020880 10.1146/annurev-med-050911-161504

[CIT0047] Menzies L Ooi C Kamath S Suckling J McKenna P Fletcher P Bullmore E Stephenson C (2007) Effects of gamma-aminobutyric acid-modulating drugs on working memory and brain function in patients with schizophrenia. Arch Gen Psychiatry 64:156–167. 17283283 10.1001/archpsyc.64.2.156

[CIT0048] Mico U Bruno A Pandolfo G Maria Romeo V Mallamace D D’Arrigo C Spina E Zoccali RA Muscatello MR (2011) Duloxetine as adjunctive treatment to clozapine in patients with schizophrenia: a randomized, placebo-controlled trial. Int Clin Psychopharmacol 26:303–310. 21934625 10.1097/YIC.0b013e32834bbc0d

[CIT0049] Mulholland C Lynch G King DJ Cooper SJ (2003) A double-blind, placebo-controlled trial of sertraline for depressive symptoms in patients with stable, chronic schizophrenia. J Psychopharmacol 17:107–112. 12680747 10.1177/0269881103017001713

[CIT0050] Pantelis C Lambert TJ (2003) Managing patients with “treatment-resistant” schizophrenia. Med J Aust 178: 62–66. 10.5694/j.1326-5377.2003.tb05310.x 12720525

[CIT0051] Passani MB Blandina P (1998) Cognitive implications for H3 and 5-HT3 receptor modulation of cortical cholinergic function: a parallel story. Methods Find Exp Clin Pharmacol 20:725–733. 9922988 10.1358/mf.1998.20.8.487510

[CIT0052] Petit M (1994) Antidepressive drug treatment of the schizophrenic subject. Encephale 20:667–674. 7895634

[CIT0053] Pitsikas N Borsini F (1996) Itasetron (DAU 6215) prevents age-related memory deficits in the rat in a multiple choice avoidance task. Eur J Pharmacol 311:115–119. 8891590 10.1016/0014-2999(96)00586-9

[CIT0054] Poyurovsky M Pashinian A Gil-Ad I Maayan R Schneidman M Fuchs C Weizman A (2002) Olanzapine-induced weight gain in patients with first-episode schizophrenia: a double-blind, placebo-controlled study of fluoxetine addition. Am J Psychiatry 159:1058–1060. 12042201 10.1176/appi.ajp.159.6.1058

[CIT0055] Poyurovsky M Isaacs I Fuchs C Schneidman M Faragian S Weizman R Weizman A(2003a) Attenuation of olanzapine-induced weight gain with reboxetine in patients with schizophrenia: a double-blind, placebo-controlled study. Am J Psychiatry 160:297–302. 12562576 10.1176/appi.ajp.160.2.297

[CIT0056] Poyurovsky M Koren D Gonopolsky I Schneidman M Fuchs C Weizman A Weizman R(2003b) Effect of the 5-HT2 antagonist mianserin on cognitive dysfunction in chronic schizophrenia patients: an add-on, double-blind placebo-controlled study. Eur Neuropsychopharmacol 13:123–128. 12650957 10.1016/s0924-977x(02)00155-4

[CIT0057] Rizk P Salazar J Raisman-Vozari R Marien M Ruberg M Colpaert F Debeir T (2006) The alpha2-adrenoceptor antagonist dexefaroxan enhances hippocampal neurogenesis by increasing the survival and differentiation of new granule cells. Neuropsychopharmacology 31:1146–1157. 16292321 10.1038/sj.npp.1300954

[CIT0058] Rizos EN Rontos I Laskos E Arsenis G Michalopoulou PG Vasilopoulos D Gournellis R Lykouras L (2008) Investigation of serum BDNF levels in drug-naive patients with schizophrenia. Prog Neuropsychopharmacol Biol Psychiatry 32:1308–1311. 18502013 10.1016/j.pnpbp.2008.04.007

[CIT0059] Rummel C Kissling W Leucht S (2006) Antidepressants for the negative symptoms of schizophrenia. Cochrane Database Syst Rev DOI:10.1002/14651858.CD005581.pub2. 10.1002/14651858.CD005581.pub2 PMC9804000 16856105

[CIT0060] Rogóz Z Skuza G Legutko B (2005) Repeated treatment with mirtazapine induces brain-derived neurotrophic factor gene expression in rats. J Physiol Pharmacol 56:661–671. 16391422

[CIT0061] Salokangas R Saarijarvi S Taiminen T Kallioniemi H Lehto H Niemi H Tuominen J Ahola V Syvälahti E (1996) Citalopram as an adjuvant in chronic schizophrenia: a double-blind placebo-controlled study. Acta Psychiatr Scand 94:175–180. 8891083 10.1111/j.1600-0447.1996.tb09844.x

[CIT0062] Schutz G Berk M (2001) Reboxetine add on therapy to haloperidol in the treatment of schizophrenia: a preliminary double-blind randomized placebo-controlled study. Int Clin Psychopharmacol 16:275–278. 11552770 10.1097/00004850-200109000-00004

[CIT0063] Shiloh R Zemishlany Z Aizenberg D Valevski A Bodinger L Munitz H Weizman A (2002) Mianserin or placebo as adjuncts to typical antipsychotics in resistant schizophrenia. Int Clin Psychopharmacol 17:59–64. 11890187 10.1097/00004850-200203000-00003

[CIT0064] Silver H Nassar A (1992) Fluvoxamine improves negative symptoms in treated chronic schizophrenia: an add-on double-blind, placebo-controlled study Biol Psychiatry 31:698–704. 1599987 10.1016/0006-3223(92)90279-9

[CIT0065] Silver H Barash I Aharon N Kaplan A Poyurovsky M (2000) Fluvoxamine augmentation of antipsychotics improves negative symptoms in psychotic chronic schizophrenic patients: a placebo-controlled study. Int Clin Psychopharmacol 15:257–261. 10993127 10.1097/00004850-200015050-00002

[CIT0066] Singh SP Singh V Kar N Chan K (2010) Efficacy of antidepressants in treating the negative symptoms of chronic schizophrenia: meta-analysis. Br J Psychiatry 197:174–179. 20807960 10.1192/bjp.bp.109.067710

[CIT0067] Siris SG Morgan V Fagerstrom R Rifkin A Cooper TB (1987) Adjunctive imipramine in the treatment of postpsychotic depression. A controlled trial. Arch Gen Psychiatry 44:533–539. 3555386 10.1001/archpsyc.1987.01800180043008

[CIT0068] Siris SG Bermanzohn PC Gonzalez A Mason SE White CV Shuwall MA (1991) The use of antidepressants for negative symptoms in a subset of schizophrenic patients. Psychopharmacol Bull 27:331–335. 1775607

[CIT0069] Solé B Jiménez E Martinez-Aran A Vieta E (2015) Cognition as a target in major depression: new developments. Eur Neuropsychopharmacol 25:231–247. 25640673 10.1016/j.euroneuro.2014.12.004

[CIT0070] Spina E De Domenico P Ruello C Longobardo N Gitto C Ancione M Di Rosa AE Caputi AP (1994) Adjunctive fluoxetine in the treatment of negative symptoms in chronic schizophrenic patients. Int Clin Psychopharmacol 9:281–285. 7868850 10.1097/00004850-199400940-00007

[CIT0071] Stahl SM (2015) Modes and nodes explain the mechanism of action of vortioxetine, a multimodal agent (MMA): enhancing serotonin release by combining serotonin (5HT) transporter inhibition with actions at 5HT receptors (5HT1A, 5HT1B, 5HT1D, 5HT7 receptors). CNS Spectr 20:93–97. 25831967 10.1017/S1092852915000139

[CIT0072] Stenberg JH Terevnikov V Joffe M Tiihonen J Tchoukhine E Burkin M Joffe G (2010) Effects of add-on mirtazapine on neurocognition in schizophrenia: a double-blind, randomized, placebo-controlled study. Int J Neuropsychopharmacol 13:433–441. 19941694 10.1017/S1461145709990897

[CIT0073] Stenberg JH Terevnikov V Joffe M Tiihonen J Burkin M Tchoukhine E Joffe G (2011) More evidence on proneurocognitive effects of add-on mirtazapine in schizophrenia. Prog Neuropsychopharmacol Biol Psychiatry 35:1080–1086. 21402120 10.1016/j.pnpbp.2011.03.004

[CIT0074] Stryjer R Rosenzcwaig S Bar F Ulman AM Weizman A Spivak B (2010) Trazodone for the treatment of neuroleptic-induced acute akathisia: a placebo-controlled, double-blind, crossover study. Clin Neuropharmacol 33:219–22. 20838215 10.1097/WNF.0b013e3181ee7f63

[CIT0075] Sumiyoshi T Park S Jayathilake K Roy A Ertugrul A Meltzer HY (2007) Effect of buspirone, a serotonin1A partial agonist, on cognitive function in schizophrenia: a randomized, double-blind, placebo-controlled study. Schizophr Res 95:158–168. 17628435 10.1016/j.schres.2007.06.008

[CIT0076] Tatara A Shimizu S Shin N Sato M Sugiuchi T Imaki J Ohno Y (2012) Modulation of antipsychotic-induced extrapyramidal side effects by medications for mood disorders. Prog Neuropsychopharmacol Biol Psychiatry 38:252–259. 22542492 10.1016/j.pnpbp.2012.04.008

[CIT0077] Terevnikov V Stenberg JH Tiihonen J Joffe M Burkin M Tchoukhine E Joffe G (2011) Add-on mirtazapine improves depressive symptoms in schizophrenia: a double-blind randomized placebo-controlled study with an open-label extension phase. Hum Psychopharmacol Clin 26:188–193. 10.1002/hup.1189 21469215

[CIT0078] Vernon JA Grudnikoff E Seidman AJ Frazier TW Vemulapalli MS Pareek P Goldberg TE Kane JM Correll CU (2014) Antidepressants for cognitive impairment in schizophrenia: a systematic review and meta-analysis. Schizophr Res 159:385–394. 25240772 10.1016/j.schres.2014.08.015 PMC4252251

[CIT0079] Wadenberg ML Wiker C Svensson TH (2007) Enhanced efficacy of both typical and atypical antipsychotic drugs by adjunctive alpha2 adrenoceptor blockade: experimental evidence. Int J Neuropsychopharmacol 10:191–202. 16707032 10.1017/S1461145706006638

[CIT0080] Waehrens J Gerlach J (1980) Antidepressant drugs in anergic schizophrenia. A double-blind cross-over study with maprotiline and placebo. Acta Psychiatr Scand 61:438–444. 6105762 10.1111/j.1600-0447.1980.tb00882.x

[CIT0081] Weiner E Ball MP Buchholz AS Gold JM Evins AE McMahon RP Buchanan RW (2012) Bupropion sustained release added to group support for smoking cessation in schizophrenia: a new randomized trial and a meta-analysis. J Clin Psychiatry 73:95–102. 21535998 10.4088/JCP.10m06143gre

[CIT0082] Whitehead C Moss S Cardno A Lewis G Furtado VA (2012) Antidepressants for people with both schizophrenia and depression (review). Cochrane Database Syst Rev DOI: 10.1002/14651858.CD002305. 10.1002/14651858.CD002305 PMC6669259 12076447

[CIT0083] Wynchank D Berk M (2003) Efficacy of nefazodone in the treatment of neuroleptic-induced extrapyramidal side effects: a double-blind randomised parallel group placebo-controlled trial. Hum Psychopharmacol Clin 18:271–275. 10.1002/hup.476 12766931

[CIT0084] Zhang ZJ Kang WH Li Q Wang XY Yao SM Ma AQ (2006) Beneficial effects of ondansetron as an adjunct to haloperidol for chronic, treatment-resistant schizophrenia: a double-blind, randomized, placebo-controlled study. Schizophr Res 88:102–110. 16959472 10.1016/j.schres.2006.07.010

[CIT0085] Zink M Englisch S Meyer-Lindenberg A (2010) Polypharmacy in schizophrenia. Current Opinion in Psychiatry 23:103–111. 20051861 10.1097/YCO.0b013e3283366427

[CIT0086] Zisook S Kasckow JW Golshan S Fellows I Solorzano E Lehman D Mohamed S Jeste DV (2009) Citalopram augmentation for subsyndromal symptoms of depression in middle-aged and older outpatients with schizophrenia and schizoaffective disorder: a randomized controlled trial. J Clin Psychiatry 70:562–571. 19192468 10.4088/jcp.08m04261

[CIT0087] Zoccali R Muscatello MR Cedro C Neri P La Torre D Spina E Di Rosa AE Meduri M (2004) The effect of mirtazapine augmentation of clozapine in the treatment of negative symptoms of schizophrenia: a double-blind, placebo-controlled study. Int Clin Psychopharmacol 19:71–76. 15076014 10.1097/00004850-200403000-00003

